# Event and Comment

**Published:** 1918-11

**Authors:** 


### DENTAL REGISTER

Vol. LXXII NOVEMBER, 1918 No. 11

### EVENT AND COMMENT

**Standardization of State Licensing Examinations**

President Logan in addressing the House of Delegates of the National Dental Association at the annual meeting makes an appeal for the Dental Educational Council of America to undertake the classification of the laws governing the licensing of dentists for practice in the various states and of the administration of these laws by the various boards of examiners. His suggestion of the possibility of classifying the various boards of examiners on the basis of their fitness for service, much the same as the dental colleges have been classified for similar ends, makes a somewhat complicated problem. Just how the Educational Council would proceed to do this is not clear from President Logan's address, but it is obvious that the same method of classification could not be used, for the reason that these bodies are made up of officials appointed by the state; and they are not acting as individuals, or as members of the dental profession, but as state officers, and their duties are defined by statutory law. They could not be classified on any basis of personal qualification, because it would be impracticable to classify any board of five dentists with a definite rating as to their individual fitness, or even as to the qualification of the entire board to pass on the credentials of a newly graduated dentist. Besides this the members of these boards are constantly changing in personnel. An efficient board this year may change very markedly within a year, and any new man coming into the board may have some ideals or personal methods which might change the policy or standing of the board very decidedly. Colonel Logan's suggestion is "that the Educational Council investigate the different methods employed by the examining boards to ascertain the professional fitness of candidates for license to practice dentistry, and from this investigation grade the boards according to their several showings in thoroughness much as the dental colleges have been classified." We know of no precedent for such a procedure and we doubt very much if the State Boards of Dental Examiners throughout the country will take kindly to such an innovation. Colonel Logan also suggests in this address the desirability of a National Board of Dental Examiners. This has been suggested before as a way to bring about reciprocity between states, but there has not yet appeared any practicable method of circumventing the fundamental principle of state sovereignty. So long as this difficulty stands in the way we can not see how any purely professional body like the National Dental Association or the Dental Educational Council can expect to establish a system of control, except by the consent either of the college or state board examinations, so far as their business methods are concerned. The members of our state boards are in a sense state officers, and it is not likely they would willingly submit to outside interference. It is possible to coerce the colleges to a certain extent, but it would not be practicable to dictate the business methods or educational standard of any state board especially if the statutes do not support such regulations. Would it not be wiser to have the Educational Council make an effort to harmonize and standardize our state dental laws?

**Army and Navy Dental Service** These extracts from President Wm. H. G. Logan's address at the annual meeting of the National Dental Association may serve to acquaint the dental profession with the main facts in the development of the dental service program for dental care of our soldiers and sailors in the great war.

*Committee on Dentistry*—April 8, 1917, the Medical Committee of the Council of National Defense created a number of committees to have charge of the various medical needs involved in the creation of a great army for overseas service, and one of these committees was a committee on dentistry; which has had large responsibilities and has done a great work in taking care of all dental activities.

*Enlisted Dental Reserve Corps*—October 6, 1917, the Senate of the United States enacted a dental law for army dental service in which was a clause specifying that dental students should be included in the law governing medical students, which permitted them to remain in college until graduation rather than to be sent abroad for immediate service. If this act had not been passed it would assuredly have come to pass in the next two or three years that there would not be sufficient dentists left to care for the civilian dental needs in our own country.

*Quota of Dentists in the Army*—At the present time one dentist is authorized to every one thousand soldiers or sailors. It is clearly evident to the dental profession that more than this proportion of dentists will be needed to properly care for the great enlistment that is now in progress. How soon we may expect a change in the proportion of dentists to enlisted soldiers will be determined very largely by the demonstration made by our army dental surgeons.

EDUCATIONAL MATTER

*Special Courses of Instruction*—During May and June, 1917, many dental colleges gave special instructions to dentists who were volunteering for dental service in the American army, and some schools have given these courses again this year.

March 15, 1918, a school for training army dental officers was instituted at Ft. Oglethorpe, for special training of dentists to serve in the army. A curriculum of study and experimental practice for two months on such dental operations as have been reported. The effort is to harmonize the work done in the fighting field as well as to improve the capacity of the men sent over.

Dental surgeons are also being attached to medical units because of their ability to devise technical treatment for many of the severe head disasters.

June 15, 1917, a special surgical head unit, was organized to be made up from the Dental Corps, which it is expected will assist materially to perfect the surgical treatment especially at the base hospitals.

*Dental Legislation*—October 6, 1917, Congress passed a law giving dentists the same rank and pay as members of the medical corps were receiving. This law gave to the Dental Corps, nine colonels, seventeen lieutenant colonels, sixty-seven majors, and a proper proportion of captains and first lieutenants. June 30, 1918 the navy dental bill was passed which gives rank and pay increases to dentists enlisted in this service. At the present time the number of navy officers has not yet been determined.

The Provost Marshal General has accepted and approved the work of the Preparedness League of American Dentists in giving free dental services to enlisted men who had defective teeth, and desires that this work be still carried on to prepare men who need such service to pass the preliminary physical examinations.

*Commissioned Dentists*—During the sixteen months since the declaration of war by the United States, the number of dental officers has increased from fifty-two to four hundred and forty-eight in the Navy; and from eighty-six to two hundred and eleven in the regular army; in the National Guard from none to two hundred and fifty-nine; in the Dental Reserve Corps from none to five thousand, five hundred and four. This totals 5,974 dentists now regularly enlisted in the army and navy. This means more dentists are now in our army than in all the other armies of the "Allies."

The commission of first lieutenant has been offered to nearly six thousand dentists and only one hundred and thirty-nine declined. All dentists accepting service must enter as lieutenants and be promoted to captains and majors. At the present time there are only 250 captains and sixty-five majors, notwithstanding 2,118 captains and 1,265 majors are authorized.

